# Pharmaceutical Household Waste Practices: Preliminary Findings from a Case Study in Poland

**DOI:** 10.1007/s00267-019-01174-7

**Published:** 2019-05-10

**Authors:** Justyna Rogowska, Agnieszka Zimmermann, Agnieszka Muszyńska, Wojciech Ratajczyk, Lidia Wolska

**Affiliations:** 1 Department of Environmental Toxicology, Faculty of Health Sciences, Medical University of Gdańsk, Dębowa Str. 23A, Gdańsk, 80-204 Poland; 2 Department of Medical and Pharmacy Law, Chair of Social Medicine, Faculty of Health Sciences, Medical University of Gdańsk, Tuwima Str. 15, Gdańsk, 80-210 Poland

**Keywords:** Pharmaceutical compounds, Management of pharmaceutical waste, Consumer behaviour, Environmental health

## Abstract

Pharmaceutical consumption continues to grow constantly. Unused/expired pharmaceuticals are disposed of to the municipal sewage system or waste disposal. Consequently, many countries have implemented a system of collecting pharmaceutical waste, with pharmacies playing an important role. It is important to educate consumers on rational consumption and the appropriate disposal of unused/expired pharmaceuticals and to identify the level of public awareness. Two studies were conducted in Poland to estimate the problem of collection and disposal of expired/unused pharmaceuticals. The purpose of the Survey I was to identify the scale of pharmaceutical consumption and the way pharmaceuticals are disposed of by various social groups. The Survey II was aimed to identify patients’ attitudes regarding expired/unused pharmaceuticals at home. Of the respondents who participated in in Survey I, almost 74% indicated that analgesics were among the over-the-counter drugs they purchased. Group of pharmaceuticals 65% of the respondents purchased were medicines for treating flu symptoms. Almost 68% of the respondents said they usually disposed of expired pharmaceuticals in their household waste or by flushing them down the toilet. In Survey II more than 35% reported that they disposed of pharmaceuticals in the same ways. Of all respondents, ~30% returned their expired pharmaceuticals to pharmacies. Most respondents (over 65%) who participated Survey I indicated that they were aware that pharmaceutical waste can be returned to pharmacies. It should be noted that local governments are currently not obliged by law to work with or compensate pharmacies in the collection and proper disposal of unused pharmaceuticals.

## Introduction

The European Federation of Pharmaceutical Industries and Associations (EFPIA) states that the pharmaceutical industry is one of the most prosperous industry sectors worldwide (EFPIA 2016). In recent years, the production of pharmaceuticals has increased significantly because of their use in aquaculture and livestock farming in the prevention and cure of fish and livestock diseases (EEA 2010; Wang and Wang 2016). This is mainly caused by a growing number of people that need health care. In Europe, around 4000 varieties of active medical substances are used. They include painkillers and anti-inflammatory drugs, antibiotics, lipid regulators, diuretics, hormonal agents, and steroids. A great proportion of those medicines is available without prescription (Arnold et al. 2014; Barrenberg and Garbe 2015). As an example, between 2002 and 2012 the consumption of metformin and ibuprofen in Germany increased from 390 t in 2002 to 1200 t in 2012 for metformin and from 250 t to 975 t for ibuprofen, respectively (Kuster et al. 2014). France, Germany and the United Kingdom account for 46% of the market volume in tonnes of active ingredients, followed by Spain, Russia and Italy. Turkey, France and Russia have the highest antibiotics consumption, followed by Italy, Spain, Germany and the United Kingdom (EEA 2010).

As a result of the growth of pharmaceuticals consumption, the environment becomes more contaminated with pharmaceutical substances and products of their degradation. The entities that produce and trade pharmaceuticals on a wholesale and retail basis must meet restrictive obligations related to the safe disposal of that waste (Zimmermann et al. 2011). At the consumer level, however, unused and used (consumed and subsequently excreted) pharmaceuticals are commonly disposed of directly to municipal sewage systems and municipal landfills (Tong et al. 2011).

Pharmaceuticals are deposited in the environment in the form of municipal solid waste, or sewage (solid and liquid), and as a gaseous waste being the result of burning in home furnace fireplaces. Burning pharmaceuticals in home furnaces, releases harmful substances into the air and then, either onto the ground as dry deposition, or into water, fog and snow as wet deposition. The active compounds included in pharmaceuticals pose threats to the ecosystem through their continuous accumulation, their toxic and persistent nature and the potential for such pollution to develop drug-resistant microbial strains (Pal 2018). Unused or expired pharmaceuticals are therefore most frequently disposed of either to municipal solid waste landfill or to the sewage system. Pharmaceuticals from individual households are transported to sewage treatment plants with the urban wastewater. This is the consequence of pharmaceuticals being excreted by persons that consume those preparations or by the disposal of unwanted or leftover pharmaceuticals into the sewage system. Depending on their properties, pharmaceuticals may be excreted in unchanged or changed forms asmetabolites. For example, ~72% of administered carbamazepine is absorbed, while 25% is in unchanged form subsequently excreted with the faeces and 2–3% of the administered dose is discharged via urine (Mohapatra et al. 2014). The results of analytical studies indicate that treatment processes applied at water treatment plants (WTPs) are usually not capable of removing pharmaceuticals and their degradation products from the wastewater (Guerra et al. 2014; Petrie et al. 2014; Du et al. 2014; Evgenidou et al. 2015). For example, studies have shown that about 50% of atenolol, metoprolol, and propranolol can be removed, up to 16.3% of carbamazepine, and between 10 and 60% of diclofenac (Roberts et al. 2016; Sun et al. 2014).Other studies show that the efficiency of diclofenac removal during sewage treatment processes is up to 80%; and for ibuprofen it is 99.7% (Yu et al. 2013; Blair et al. 2015). Threats to the environment result from physicochemical properties of compounds, which are components of pharmaceuticals, make them bioavailable and toxic. In addition, they can cause more potent environmental effects than other contaminants, because they are designed to elicit specific biological effects at relatively low concentrations. These specific effects can be expressed as behavioural, physiological and histological alterations (Arnold et al. 2014). Possible bioaccumulation and transmission in the food chain may additionally cause a threat to wildlife. For example, diclofenac accumulates in the bile of rainbow trout by a factor of between 509 ± 27 and 657 ± 25 and can interfere with the biochemical functions of fish and lead to tissue damage (Mehinto et al. 2010).

Pharmaceuticals can migrate to drinking water (WHO 2011). About 60% of the drinking water in the European Union (EU) comes from groundwater, another 40% from other sources, including surface waters. Data from 2014 received from the National Sanitary Inspection in Poland indicates that 71.2% of water supplied to inhabitants came from underground water intakes and 28.8% from surface water intakes (SSI 2015). Freshwater resources per inhabitant are considered an important indicator for measuring the sustainability of water resources. Among the EU Member States countries which experience ‘water stress’ are Poland, Czech Republic, Cyprus and Malta (EUROSTAT 2017).

The contamination of groundwater with pharmaceuticals can cause negative consequences for human health. For example, analysis of groundwater samples from selected metropolitan areas of Barcelona, Spain, carried out by López-Serna et al. (2013), indicated concentrations above 100 ng/L for many compounds, especially analgesics and antibiotics, and even higher than 1 μg/L for two macrolide antibiotics: spiramycin and azithromycin (López-Serna et al. 2013). In another study that undertook chemical analyses of drinking water in France, 25 compounds (out of 51 tested for) were present; and the most frequently detected was salicylic acid. Carbamazepine and the β-blocker atenolol were also found in smaller quantities (up to 2 ng/L) in more than 30% of the contaminated drinking waters (Vulliet et al. 2011).

Therefore, legislation regarding the management of pharmaceutical waste has been approved in many countries to protect human health and the environment (Voudrias et al. 2012). According to the Directives 2001/83/EC and 2001/82/EC each EU member state must establish a system of collecting expired or unused pharmaceuticals. In Sweden the unused pharmaceuticals should be returned to pharmacies. The pharmacies provide households with special transparent plastic bags which are to be used to hold unused drugs for eventual return to a pharmacy. The returned pharmaceuticals are then placed in sealed boxes and transported to specially designated incineration stations by wholesalers where they are subsequently burned at high temperatures under supervision and the emitted gas is purified before release into the air. The system is financed by the state budget (Persson et al. 2009). A similar system is implemented in Lithuania, where the residual (unsold, outdated) pharmaceuticals are returned to their producers or to the pharmaceutical waste management company (Kruopienė and Dvarionienė 2010). However, Kruopienė and Dvarionienė (2010) report that this system is not working properly yet. In consequence, over 80% of household unused, unwanted or expired pharmaceuticals are disposed of via household waste (Kruopienė and Dvarionienė 2010).

In Austria patients can return unused pharmaceuticals to the pharmacies. Additionally, pharmaceuticals can also be handed over to a public waste collection point. In Vienna, the capital city of Austria, public collection points are maintained by the municipality of Vienna (Vogler et al. 2014). The Republic of Serbia is in process of aligning with the health laws of the EU. Medical waste management rules adopted in 2010, impose an obligation for pharmacies to take unused and expired pharmaceuticals from the public, keep them as pharmaceutical waste and return them to wholesalers, manufacturers or special operators trained to collect and transport the waste for destruction. However, because these regulations are not clear, it is difficult to determine which entity should pay the related costs (Kusturica et al. 2012). Information obtained by Vollmer 2010 shows that nearly all countries in Europe responded that this waste must be returned to pharmacies; some countries also provided for public collection points (Vollmer 2010).

These systems are not able to fulfill their role without proper consumer (patients) behaviour. Preventive actions that eliminate the waste prior to disposal are more important. Immediate action should be taken to minimise the use of pharmaceuticals by educating consumers on their correct and rational consumption and the appropriate disposal of unused and/or expired pharmaceuticals. The effectiveness of those actions is mainly dependent on the awareness of the society. Therefore, the first stage of preventive actions should be to identify the level of public awareness. The proper behaviour of patients is primarily influenced by their awareness of the environmental and health impacts effects of inappropriate processing with unused/expired pharmaceuticals. Review research carried out by Tong et al. (2011) and Kusturica et al. (2017) suggest that patients still very often lack the knowledge of the proper disposal of their unused medication and how improper disposal methods may affect the environment.

The aim of our research was to obtain information about the use of pharmaceuticals and the disposal of unused and expired pharmaceuticals by residents of Poland. Similar surveys were carried out in Serbia, Great Britain, Lithuania and Sweden, among others. Research conducted by Tong et al. (2011) and Kusturica et al., (2017) aimed to determine the practice of medication disposal around the world and get insight into possible association between environmental awareness and people’s behaviour. This research did not include data from Poland, because in peer-reviewed journals, there is no information about the practice of Polish consumers. In our research, we decided to fill this gap.

## Materials and Methods

### Description of Research Area

The Polish market of medicines is one of the largest in Europe. Poland is recognised as a market with a significant growth trend. A major proportion of that market is comprised of medicines and food supplements sold directly, i.e., without prescription and over-the-counter (OTC), and these are among the fastest developing groups of products (IBGR 2014). IMS Institute for Healthcare Informatics (now IQVIA) estimates that the Polish market is likely to grow at 4% per annum. Hospitals using medicines in contagious and oncological disease treatment form the main driving force of that growth (IMS 2014). In addition, the structure of the sale of pharmaceuticals by therapeutic groups has changed. In 2000, medicines for the treatment of cardiovascular system problems were at the top of the list of the most frequently purchased medicines, while medicines for the treatment of nervous system or digestive tract and anti-infective drugs were farther down the list. By 2014, oncological medicines were at the top of the list and they were followed by medicines for the treatment of cardiovascular, digestive, respiratory system disorders as well as antidiabetics (IMS 2014). Statistical surveys carried out in 2016 by the Public Opinion Research Center (CBOS) with a group of 981 Polish residents showed that in the preceding 12 months OTC medicines and dietary supplements were used by almost nine out of ten adults (89%). Most often these were painkillers and anti-inflammatories (68%), and products to alleviate the symptoms of colds or flu (68%). More than half of the respondents (52%) took vitamins, minerals and other agents that improve the body’s general immunity during this 12 month period. Over a quarter of the adults surveyed (27%) used over-the-counter medications to alleviate digestive disorders (CBOS 2016).

Together with the growth in sales and consumption of medicines, the volume of unused and expired pharmaceuticals which is disposed of by consumers has also increased.

Unused and expired pharmaceuticals, as well as pharmaceuticals that have been recalled as dangerous wastes are defined by Polish Law (Art. 3.4 of the Waste Act of 14 December 2012 (Journal of Laws No. 2016.1987)). They can be ‘toxic’ (and ‘very toxic’), ‘harmful’, ‘irritating’, ‘carcinogenic’, ‘endocrine’ and ‘mutagenic’. The collection and disposal of hazardous pharmaceutical waste is ad hoc. Some municipal offices, health care centres or pharmacies voluntarily dispose of hazardous waste in special containers. But the collection of the containers and the safe disposal of their contents is inconsistently managed—sometimes collected by pharmaceutical wholesalers (as a courtesy), otherwise especially in small towns, there is no guarantee of collection and safe disposal (Zimmermann et al. 2011).

Pharmaceuticals from patients are treated as municipal solid waste, because they are household waste. Municipalities are obliged by law to arrange for the collection of municipal solid waste from the owners of residential properties (Art. 6c.1 of the Act on Maintaining Municipalities Clean and in Order of 13 September 1996, i.e. Journal of Laws No. 2016.250, as amended). Unfortunately, there are no precise regulations that would impose an absolute obligation for the segregation of pharmaceuticals by municipalities. The Act does not provide any instructions in their internal regulations to municipalities (being the source of local law) outlining their obligations for the special handling of pharmaceuticals. The regulations set forth requirements for maintaining the cleanliness of facilities and orders regarding the selective collection of expired medicines from households (Art. 4.1 in conjunction with Section 2.1a). Some pharmacies take part in the process on a voluntary basis; and as such they are acting for the benefit of the local community and fulfilling their social responsibility standards aimed at health protection. However, there are places where local governments do not pay for the purchase and maintenance of pharmaceutical waste containers for pharmacies. This is particularly the case in rural sites and small towns. Thus, patients are often not able to leave expired pharmaceuticals at pharmacies. Expired pharmaceuticals coming from patients are not supervised by sanitary authorities. For example, the case of narcotic drugs is a particular concern, especially when unused narcotics then become available for illegal trading. Oncological medicines, which can constitute a potential threat to the environment, are also a matter of concern. There are no legal regulations that would control such instances. Pharmacies are not formally involved in the disposal process nor are they legally required to take up any actions, and at the same time, local governments do not have legal tools for that purpose. It seems that pharmacies do collect those pharmaceuticals from patients on a voluntary basis and then utilise them or dispose of them safely, demonstrate their involvement in and care of the environment of the local community despite of the lack of related legal regulations. Such an attitude should be promoted by state policy.

In order to acquire information about the use of pharmaceuticals and the disposal of unused and expired pharmaceuticals, two surveys were carried out: Survey I concerned the use of pharmaceuticals and the disposal of household pharmaceuticals, Survey II focused on the disposal of expired household pharmaceuticals by persons visiting pharmacies, where expired medicines can be left despite of the lack of formal cooperation with local governments. Both surveys were conducted during 2015.

### Characteristic of Surveys

For Survey I, which was to determine consumption quantities and the methods of disposal of unused and expired pharmaceuticals by various groups, we created a digital questionnaire for distribution by email. In order to reach people from a variety of regions of Poland, when distributing the questionnaire by email, we used the ‘snowball’ method of survey sample selection. The survey was sent to a random selection of students and asked them to complete the questionnaire, and to forward copies of the blank questionnaire to other people on their own contact lists who might also take part. We were aware that by using this method there would be a problem with the final representativeness of the sample, but one of the counterbalancing advantages of this method is that it can be used to carry out qualitative research. Thanks to this survey methodology we obtained a lot of valuable information. As a result of the questionnaire being forwarded to third parties, our questions reached people in several parts of Poland that would have been impossible for us to reach with a different method. The questionnaire was voluntary and anonymous. The questionnaire included 27 questions:

- 21 fact-related questions, including 17 questions concerning pharmaceuticals and 4 questions concerning the disposal of expired pharmaceuticals;
- 6 questions that helped to define the demographic profile of participants and that were not related to facts concerning pharmaceutical consumption and disposal.

Survey I encompassed the entire geographic territory of Poland. For the purpose of the research, the analysis covered only those questions in the questionnaire that were related to the volumes of pharmaceuticals consumed and the disposal of unused and/or expired pharmaceuticals.

In Survey II, the researchers attempted to identify patients’ attitudes to expired/unused pharmaceuticals that are found in the home medicine cabinet; and their opinions on the role of a generally accessible pharmacy in the selective collection of those medicines. The questionnaire was addressed to adult customers of various types of pharmacies (pharmacies located in housing estates, outpatient clinics, hospitals, shopping centres) where expired medicines may be left in special containers. Customers put their completed questionnaires in boxes located in pharmacies or left them with the pharmacist. During the research, 1000 surveys were distributed, and 635 completed questionnaires were returned (63.5% return rate). Nonprobability sampling was used. The study was designed for pharmacy customers. Survey II was conducted in the Pomeranian Voivodeship (District) of northern Poland. In this area there are places where no formal cooperation has been established between local governments and pharmacies for the collection and disposal of unused and expired medical products. The region was also selected for our study because it coincided with, and was expected to benefit from, an environmental, social awareness program focused on waste disposal: the Regional Operational Program of the Pomorskie Voivodeship for the years 2014–2020. The population of the District is approximately 1,600,000 adults. Survey II was conducted concurrently with Survey I, and was voluntary and anonymous. Written consent to use their premises and cooperation in the survey was obtained from the participating owners and managers of pharmacies. The questionnaire was comprised of 18 questions (including three respondent demographic questions). We preceded this survey with a pilot survey conducted with a small volunteer study population of 22 adult persons.

All statistical calculations were undertaken using the statistical analysis package StatSoft. Inc. STATISTICA (data analysis software system) version 10 and recorded in Excel spreadsheets. We determined the interrelationships between qualitative variables using Chi^2^ tests. In all calculations, the level of significance was *p* = 0.05.

## Results and discussion

In Survey I, 450 people participated, including 306 women (68%) and 144 men (32%). The study population consisted of the following age groups: 78% of participants were up to 40 years of age, around 18% were between 41 and 60, and people older than 61 constituted the smallest group (over 4%). Survey II was completed by 635 people, including 461 women (72.6%) and 174 men (27.4%). Out of 633 respondents (who reported their ages), 258 (40.8%) were aged up to 39, 261 (41.2%) were between 40 and 60, and 114 (18.0%) were over 60 (2 people did not give their ages).

### Consumption of Pharmaceuticals

Survey I: Given that the OTC market represented approximately 42% of the value share of the total Polish pharmaceutical market in 2014, respondents were asked “Which OTC do you buy most often at the pharmacy?” Respondents were presented with the 5 groups of medicines that account for the highest value of pharmacy sales according to PASMI (Polish Association of the Self-Medication Industry) data: cold or influenza drugs, vitamins and minerals, analgesics, digestive aids, and food supplements (PASMI 2014).

Responses about the frequency and types of respondents’ OTC purchases indicated that most purchases (almost 72%) were of analgesics (Table 1). Another commonly purchased group of pharmaceuticals was medicines for the treatment of flu symptoms (over 65%). The respondents bought food supplements most rarely (around 17%).

**Table 1:** Distribution of answers to the question about OTC

Which OTC do you buy most often in pharmacy?	Investigated group (*n* = 450)	
	Total answers	Percentage of group (%)
Analgesics	323	71.8
Cold and flu drugs	294	65.3
Digestive drugs	70	15.6
Vitamins & minerals	186	41.3
Food supplements	78	17.3

The respondents could give several answers

**Fig. 1:**
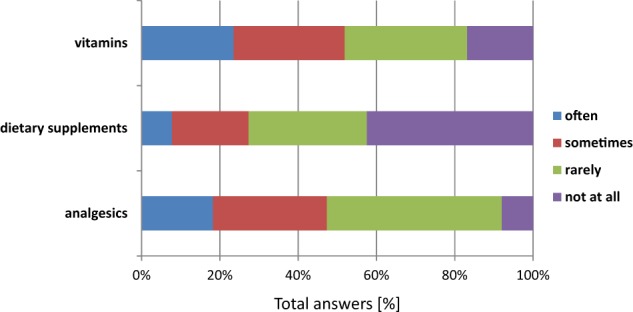
The rate of OTC pharmaceuticals use

The respondents were also asked “How often do you use OTC pharmaceuticals that are available from the pharmacy without a prescription?”. The survey gave respondents three categories to choose based on CBOS (Public Opinion Research Center) data. According to that data from 2010, which came from research conducted with a representative random sample of adults in Poland, the most commonly used over-the-counter drugs included analgesics (71.8% of respondents), cold and flu drugs (65%% of respondents), and vitamins and minerals (41.3% of respondents) (CBOS 2010). In Survey I, 74% of respondents stated that they used painkillers sometimes or rarely (Fig. 1), and mainly for severe pain. Only 6% of the respondents stated that they did not use painkillers at all. Almost 60% of the respondents buy OTC pharmaceuticals before they need them. Similar results were presented by Zorpas et al. (2018), who surveyed a group of 184 adults (44.6% men and 55.4% women) living in the District of Nicosia (Cyprus). Their study showed that painkillers were the most used by the respondents (65.8%); furthermore, they showed that 86.6% of men and 83.3% of women used the pharmacy with or without a doctor’s prescription (Zorpas et al. 2018).

In the Survey II, patients were asked about where they buy pharmaceuticals. Responses indicated that they usually bought pharmaceuticals at pharmacies (622 respondents, 98%). Respondents also mentioned other sources including online pharmacies (48 respondents, 7.6%), food shops (22.7%) and chemist’s (21.1%). Almost 15% of the respondents bought pharmaceuticals at petrol stations (Table 2).

**Table 2:** Distribution of answers to the question concerning places where the respondents bought pharmaceuticals

Please specify places where you have bought pharmaceuticals this year and during last year	Investigated group (*n* = 635)	
	Total answers	Percentage of group (%)
At a pharmacy	622	98.0
At a chemist (shop with cosmetics and sanitary articles)	134	21.1
At a food shop	144	22.7
At a petrol station	93	14.6
via the Internet – at an online pharmacy	48	7.6
via the Internet – in response to an advertisement	7	1.1
via the Internet – other	3	0.5

The respondents could give several answers

The distribution of answers to the above question was dependent on the place of residence of the participant to a statistically important extent. Pharmaceuticals were bought at the chemist’s usually by residents of large cities (test statistics: Chi^2^ = 8.02, *p* value = 0.0181). In turn, inhabitants of villages usually bought pharmaceuticals at grocery stores (test statistics: Chi^2^ = 7.80, *p* value = 0.0202).

The distribution of answers to the above question was also dependant on age to a statistically important extent. Young people up to the age of 39 bought pharmaceuticals at pharmacies much more often than other respondents (test statistic: Chi^2^ = 23.87, *p* value = 0.0001). Young persons also bought pharmaceuticals at food shops much more frequently (test statistics: Chi^2^ = 12.83, *p* value = 0.0016), at petrol stations (test statistics: Chi^2^ = 12.09, *p* value = 0.0024), or via the Internet (test statistics: Chi^2^ = 9.70, *p* value = 0.0078). Thus, the pharmacy, particularly in the case of younger persons, is not considered as the only place where pharmaceuticals are bought.

### Disposal of Household Pharmaceuticals

The purpose of the study was to find out how respondents dispose of their expired pharmaceuticals. Almost 68% of the respondents in Survey I usually disposed of expired pharmaceuticals along with their household waste or via the toilet into the sewage system. In Survey II more than 35% reported disposing of pharmaceuticals by these same methods. This proves that a significant portion of the respondent population does not know or follow the rules for the proper disposal of expired pharmaceuticals. Differences between the two sets of data will be associated with the form of the questions and in the ways the two questionnaires were distributed. Almost 68% of the respondents to Survey I said that they usually disposed of unused or expired pharmaceuticals along with their household waste or via the toilet into the sewage system. In Survey II more than 35% of the respondents reported disposing of their unused or expired pharmaceuticals by the same methods. Out of all respondents, around 30% (Survey I 135 persons—30%; Survey II 227 persons—35.7%) reported that they left expired pharmaceuticals at pharmacies. However, the weak result obtained under the Survey II is because the questionnaire was addressed to pharmacy customers and not the population in general (OTC pharmaceuticals may be bought somewhere else). Our results are comparable to results obtained for the population of Great Britain (63.2% of unused medicines are disposed of with the solid waste and 21.8% are returned to pharmacies) (Bound and Voulvoulis 2005). The analysis of the patients’ behaviour in Serbia shows that only 4% of respondents leave unused pharmaceuticals at pharmacies (Kusturica et al. 2012). Krupiene and Dvarioniene (2010) obtained similar results to the Serbian findings during studies conducted among the inhabitants of Lithuania (around 3%). In Sweden, pharmaceuticals are disposed of with solid waste only by 3% of households and 43% of households return them to pharmacies (Persson et al. 2009). Face-to-face interviews with Portugal householders showed that 69% of the respondents claimed returning pharmaceutical waste to the local pharmacy. However, in the opinion of the authors of our study, this figure is overrated, probably owing to a possible ‘good answer’ effect (Dias-Ferreira et al. 2016). Meanwhile, a cross-sectional questionnaire-based survey of 600 households was undertaken in the Ga South Municipal Assembly in Accra (Ghana) in 2014, showed that 80% of respondents indicated that unwanted medicines were discarded with household waste (Udofia et al. 2017). In Cyprus, 92.4% of respondents in another survey indicated that they disposed of unused and expired drugs along with their household waste, and 24.5% indicated they favoured disposal in the toilet, which is considered to be the second most favourable method (Zorpas et al. 2018). Similar results were obtained by Shaaban et al. (2018) in studies carried out among residents in Saudi Arabia. There, the majority of respondents (62.9%) discarded unwanted medications in household waste, with the remainder either emptying them into the sink or the toilet (16.6%), or returning them to a pharmacy (6.5%), or a physician (1.4%). However, a factor that may have influenced such results is the fact that in Saudi Arabia the government healthcare system offers free medications for all citizens (Shaaban et al. 2018). Hong Kong, one study shows that residents most frequently dispose of unused medicines in regular waste bins (53.9%) (Chung and Brooks 2019).

In addition, it must be noted that the statistical analysis based on data obtained from both our questionnaires did not show any dependent relationship between education levels and the disposal of expired/unused pharmaceuticals. This contrasted with the findings of Shaaban et al. (2018) in their research with a Saudi Arabian population. They found in that study, that respondents with higher education (58.4%) were more likely to return medications to a pharmacy than would those with lower educational levels. In addition, their study found that gender also exerted an influence on the practice of giving unwanted or unused medications to someone else. Females were more likely than males to give unwanted medications to others as a method of disposal (84.1% vs. 15.9%, respectively) (Shaaban et al. 2018). A similar finding occurred in the results from our Survey II, where the distribution of answers to the questions about the possibility of returning unused or expired medications to a pharmacy, was dependant on gender to a statistically significant extent (test statistics: Chi^2^ = 6.58, *p* value = 0.0103). In addition, we found that men, much more often than women, declared that they did not remember what had happened to their expired or unused medicines. This may indicate that they are not aware or are not interested in what happens with the pharmaceuticals in their household. That may be treated as a guideline for awareness campaigns, which should be mainly addressed to men.

Results from Survey I suggested that age was not a statistically significant predictor of disposal methods. However, a contrary result was obtained in Survey II. The answer ‘I dispose of it with solid waste or discharged to the sewage system’ was much more often chosen by young persons of up to 39 (Chi^2^ = 11.09, *p* = 0.0039). The response ‘I left it at the pharmacy’ was usually chosen by elder persons of over 60 years (Chi^2^ = 7.91, *p* = 0.0191).

### The Role of a Community Pharmacy in the Selective Collection of Pharmaceuticals

As mentioned above, in many countries, pharmacies are considered an appropriate place to ensure that proper preventative actions are taken concerning reasonable use of medicines and for providing education concerning correct segregation of expired pharmaceuticals. In both studies, the respondents were asked about the role of pharmacies in the system of collecting expired pharmaceuticals. The data presented in Table 3 indicates that over 65% of the respondents are aware that expired pharmaceuticals can be left with pharmacies in the opinion of most respondents, pharmacies should accept expired pharmaceuticals for utilisation purposes. Most respondents also believed pharmacies to be an appropriate place to leave expired pharmaceuticals.

**Table 3:** The role of a pharmacy in the selective collection of pharmaceuticals

Question	Total answers	Percentage of group (%)
The survey (I)	Investigated group (*n* = 450)	
Do you realize that expired/unused pharmaceuticals can be returned to a pharmacy for disposal?		
Yes	293	65.1
No	157	34.9
Do you think the pharmacy is obliged to accept expired/unused pharmaceuticals for the purpose of disposal?		
Yes, but only selected pharmacies	57	12.7
Yes, each pharmacy	256	56.9
No, pharmacies do not have such an obligation	16	3.5
I don’t know	121	26.9

The survey (II)	Investigated group (*n* = 635)	
Do you think that the pharmacy is a place where you should dispose of expired/unused pharmaceuticals?		
Strongly agree	415	65.4
Somewhat agree	100	15.7
I have no opinion	70	11.0
Somewhat disagree	42	6.6
Strongly disagree	8	1.3

At the same time, only around 30% follow that practice. This may result from the fact that the respondents are not aware of the harmful impact of pharmaceuticals on the environment. Because of their low environmental awareness, patients dispose of their pharmaceuticals together with their solid wastes or by pouring them into the sewage system or by burning them. This may also be a matter of convenience since there may not be an appropriate disposal site (pharmacy) near their home. This inappropriate behaviour with regard to disposal is particularly noticeable in rural sites. Over 74% of the respondents in Survey I who lived in villages admitted that they disposed expired/unused pharmaceuticals of with solid wastes or discharge them into the sewage system. Around 64% of the respondents in Survey II acted in the same way.

Survey I respondents were also asked whether a pharmacist had ever refused to accept their pharmaceuticals. Almost 5% of the respondents stated that their pharmaceuticals had not been accepted by the pharmacy. This could be because pharmacies are not obliged by law to collect these wastes (Waste Act 2012). In addition, it must be noted that the collection of expired pharmaceuticals from patients generates high costs to the pharmacy. These costs should be paid by local governments or in cooperation with pharmacies, and not paid by the pharmacy alone, because unused/expired pharmaceuticals are municipal waste. At the same time, almost 65% of the respondents admitted that they had never left pharmaceuticals with pharmacies, which implies that 30% of the respondents occasionally left expired or unused pharmaceuticals with pharmacies.

In Romania, a study has shown that there are problems with: a lack of procedure, incomplete legislation, operators exceeding their contracted costs, and the high costs of collecting expired pharmaceuticals from patients. A nationwide study by Bungau et al. (2018) with a group of 521 pharmacists, showed that 16% of the participants work for pharmacies that do not collect unused or expired drugs from the people; and nearly 33% of those surveyed have refused, at least once, to take unused medicines from people when asked (Bungau et al. 2018). However, according to another study carried out in the Republic of Serbia, in March 2013, 76.5% of pharmacies collect expired medicines that people bring to them, in order to get them put away, while 23.5% of the pharmacies surveyed assert that they do not collect pharmaceutical waste from households at all (Manojlović et al. 2015)

### Limitation of Study

Our study has some limitations. The respondents who took part in Survey II were selected because they were customers of pharmacies, i.e., people who primarily buy drugs in pharmacies (98%). By comparison, the population sampling methodology for Survey I meant that respondents were mainly young people with secondary or higher education. Differences between the results of the two surveys about unused and expired drugs have been discussed above; and in addition, we also obtained results that were similar between the two studies about, for example, the role of a pharmacy in the collection of unused or expired pharmaceuticals. In Survey II, one of the possible answers to the question concerning the disposal of pharmaceuticals at home or by leaving them at pharmacies was ‘I do not remember such a situation’ (whereas this would be an invalid answer in Survey I). In Survey II, the ‘I do not remember’ answer was given by 153 people (24.2%) and this may indicate that respondents either consumed the medicine before it expired or did not have medicines at home. However, it may also relate to the situation of some respondents knowing that burning, discharging to the sewage system, burying or otherwise disposing of expired pharmaceuticals at home is irresponsible; and perhaps, they did not want to admit that they had used any of these forms of disposal and therefore chose the ‘I do not remember’ answer in order to avoid presenting themselves in unfavourably. Regarding gender, a similar number of men and women participated in both studies. We are aware, however, that in order to accurately reproduce the gender proportions of Polish society it would be necessary to increase the proportion of male respondents in the surveys.

## Conclusion

It is difficult to imagine today’s world without pharmaceuticals. They are commonly used, and society is not able to function without them. Considering the existing environmental burden and the impairment of the environment’s regenerative capacity, there a need to develop and produce pharmaceuticals which will be less toxic for ecosystems and biodegradable (‘green drugs’). There is also a need to establish also systems for the correct segregation of pharmaceutical waste. However, first steps should be carried out to minimise the use of pharmaceuticals by educating consumers on their correct and rational consumption. Pharmacists who have direct contact with patients should play an important role in this field. We recommend education programmes for pharmacists to provide them with information about what sort of drug disposal information their consumers need. It is worth mentioning that there is an association between a survey participant’s awareness of the proper methods for disposing of pharmaceuticals and their choice of a disposal method. Studies carried out by Shaaban et al. (2018) demonstrated that respondents who received instructions about proper disposal are more likely to return pharmaceuticals to a pharmacy. At the same time 73% of the respondents declared that they had never received any such instructions.

In addition, it is recommended that patients and the population in general be educated on the risks related to pharmaceuticals that find their way into the environment; and awareness of their impact on aquatic and water-dependant ecosystems. Patients must become aware that the condition of the natural environment is directly dependent on their decisions and conduct. These actions should be taken at various levels local, regional and national and referral to various social groups. However, it should be noted that shaping social awareness is a long-term process. Although there has been a national (not-for-profit) social awareness programme running in Australia since 1998, funded by the Commonwealth Government, to address the safe disposal of medicines (RUM project), a survey conducted in 2016 with 4302 respondents, showed that less than 18% had heard of the program and that most people dispose of unwanted medicines in their household rubbish or into the sewerage (Bettington et al. 2018).

It seems that campaigns targeting small, specific communities can be more effective. A campaign by a private communication company in Turkey was an example aimed at increasing the awareness about the proper storage and use of pharmaceuticals. The campaign was targeted employees of the company and consisted of activities such as training (a symposium, a distant learning course, and information material provided to employees); a survey about drug use behaviours; and a “take-back” programme for unused drugs using a drug-box within the company, and the still-useful drugs (assessed as useable by experts) were delivered to animal shelters, and others were disposed of with least harm to the environment (Akici et al. 2018). Subsequently, the programme outcomes were assessed to see if it had changed the way people dealt with their expired or unused pharmaceuticals. Almost 50% all participants (46.5%) declared a recent change in their disposal behaviour. These participants were also significantly less likely to dispose of drugs inappropriately, practice self-medication, be unaware of expired drugs at home, or fail to store drugs according to the labelling. In addition, those exhibiting positive behaviours were shown to have a high tendency of sharing those behaviours with their contacts (Akici et al. 2018).

It seems necessary that Polish municipalities should be obliged by law to cooperate with pharmacies in the selective collection of pharmaceutical waste because the surveys show that patients believe that a pharmacy is the appropriate place to dispose of expired or unused pharmaceuticals. Therefore, we recommend that there should be cooperation in drug waste management programmes between pharmacies and provincial governments, especially in rural areas.
